# Multicenter Experience of Aortic Valve Leaflet Modification in TAVR: From the Asia-Pacific Electrosurgery Working Group

**DOI:** 10.1016/j.jacasi.2026.04.017

**Published:** 2026-06-24

**Authors:** Chun-Ka Wong, Simon Cheung-Chi Lam, Wei-Hsian Yin, Yung-Tsai Lee, Nattawut Wongpraparut, Ronen Gurvitch, Mann Chandavimol, Kent Chak-Yu So, Ka-Chun Un, Ho-On Alston Conrad Chiu, Tien-Ping Tsao, Matthew Brooks, Tawai Ngernsritrakul, Leo Kar Lok Lai, Kevin Ka-Ho Kam, Daniel Tai-Leung Chan, Gilbert H.L. Tang, Kwong-Yue Eric Chan, Karl Poon

**Affiliations:** a Cardiology Division, Department of Medicine, School of Clinical Medicine, Li Ka Shing Faculty of Medicine, The University of Hong Kong, Hong Kong; b Cardiology Division, Department of Medicine, Queen Mary Hospital, Hong Kong; c Heart Center, Cheng Hsin General Hospital, Taipei, Taiwan; d Faculty of Medicine, School of Medicine, National Yang Ming Chiao Tung University, Hsinchu, Taiwan; e Department of Exercise and Health Science, National Taipei University of Nursing and Health Sciences, Taipei, Taiwan; f Department of Medicine, Siriraj Hospital, Mahidol University, Bangkok, Thailand; g Department of Cardiology, Royal Melbourne Hospital, Melbourne, Australia; h Department of Medicine, Ramathibodi Hospital, Mahidol University, Bangkok, Thailand; i Division of Cardiology, Department of Medicine and Therapeutics, Prince of Wales Hospital, The Chinese University of Hong Kong, Hong Kong; j Li Ka Shing Institutes of Health Science, The Chinese University of Hong Kong, Hong Kong; k Faculty of Medicine, National Defense Medical University, Taipei, Taiwan; l Department of Surgery, School of Clinical Medicine, Li Ka Shing Faculty of Medicine, The University of Hong Kong, Hong Kong; m Department of Cardiothoracic Surgery, Queen Mary Hospital, Hong Kong; n Department of Cardiovascular Surgery, Mount Sinai Health System, New York, New York, USA; o Cardiac Medical Unit, Grantham Hospital, Hong Kong; p University of Queensland, Brisbane, Australia; q The Prince Charles Hospital, Metro North Health, Brisbane, Australia

**Keywords:** BASILICA, leaflet modification, transcatheter aortic valve replacement, transcatheter electrosurgery, UNICORN

## Abstract

**Background:**

Safety and efficacy of leaflet modification in transcatheter aortic valve replacement (TAVR) to reduce coronary obstruction risk have been demonstrated in North American and European populations, but real-world data from the Asia-Pacific remain limited.

**Objectives:**

The authors aimed to report the real-world procedural and clinical outcomes of bioprosthetic or native aortic scallop intentional laceration to prevent iatrogenic coronary artery obstruction (BASILICA) and undermining iatrogenic coronary obstruction with radiofrequency needle (UNICORN) leaflet modification techniques during TAVR in Asia-Pacific.

**Methods:**

This multicenter retrospective observational registry included consecutive patients undergoing aortic valve leaflet modification during TAVR across 7 centers in the Asia-Pacific from April 2019 to January 2026. Primary outcome was procedural success, defined as successful leaflet traversal, leaflet modification, and transcatheter heart valve implantation without coronary obstruction, emergent surgery, stroke, or mortality in 30 days. Secondary outcomes included 30-day major adverse events per the Valve Academic Research Consortium 3 definitions.

**Results:**

Among 100 patients, 72.2% were female with a median age of 80.0 years. BASILICA was performed in 67.0%, UNICORN in 30.0%, and BASILICA-UNICORN in 3.00%. Annual case volume increased nearly 10-fold during the study period. Index valves were predominantly small (≤21 mm) surgical bioprostheses. Procedural success was achieved in 85.1% of BASILICA patients and 96.7% of UNICORN patients. Coronary obstruction occurred in 2.99% of BASILICA patients.

**Conclusions:**

Transcatheter electrosurgical leaflet modification techniques are associated with acceptable short-term outcome in high-risk patients undergoing TAVR in the Asia-Pacific region.

Over the past 2 decades, transcatheter aortic valve replacement (TAVR) has been increasingly performed worldwide for the treatment of aortic stenosis and regurgitation. Patients at high risk of coronary obstruction, such as those with low coronary height or narrow valve to coronary or valve to sinotubular junction distance, have been deemed at high risk of sinus sequestration or coronary obstruction in TAVR.^1^ Techniques such as chimney^2^ and snorkel stenting^3^ have helped reduce the risk of coronary obstruction but may complicate future coronary access.

The bioprosthetic or native aortic scallop intentional laceration to prevent iatrogenic coronary artery obstruction (BASILICA) technique, introduced in 2018, was the first method developed for aortic leaflet modification.^4^, ^5^, ^6^ It uses electrosurgery to create a linear laceration in the leaflet adjacent to the coronary ostium, thereby improving blood flow to the coronary artery. Subsequently, the undermining iatrogenic coronary obstruction with radiofrequency needle (UNICORN) technique was developed, which involves serial dilation of a fenestration in the target leaflet, followed by intraleaflet deployment of a transcatheter heart valve.^7^, ^8^, ^9^, ^10^ This approach enables simultaneous leaflet opening, entrapment at the sides, and transcatheter heart valve deployment. More recently, mechanical leaflet splitting using dedicated devices has also become commercially available in select regions.^11^

To date, prospective trials and multicenter registries from North America^12^ and Europe^13^ have demonstrated the safety and efficacy of the BASILICA technique; however, real-world data from the Asia-Pacific region remain limited.^14^ The Asia-Pacific Electrosurgery Working Group–Aortic Valve Leaflet Modification Registry aims to analyze the real-world application of BASILICA and UNICORN in this region.

## Methods

### Study design and patient population

This multicenter, retrospective, observational registry included consecutive patients who underwent aortic valve leaflet modification during TAVR across 7 experienced centers in Hong Kong, Taiwan, Thailand, and Australia. Inclusion criteria were adults aged ≥18 years undergoing elective TAVR (both native and valve-in-valve procedures) with BASILICA or UNICORN leaflet modification. Exclusion criteria included alternative leaflet modification techniques such as surgical resection of prosthetic valve leaflets under direct vision (SURPLUS),^15^ CATHeter Electrosurgical Debulking and RemovAL (CATHEDRAL),^16^ and mechanical splitting. The study was approved by the Institutional Review Board of the University of Hong Kong and Hospital Authority Hong Kong West Cluster (HKU/HA HKW IRB; UW 12-177). Informed consent from individual patients was waived as the study involved analysis of anonymized data from only the hospital registry. The study was conducted in accordance with the Declaration of Helsinki and reported following the STROBE (Strengthening the Reporting of Observational Studies in Epidemiology) guidelines.

### Data collection

Baseline patient, computed tomography anatomy, device, and procedural characteristics were collected. Procedure details included mode of anesthesia, use of transesophageal echocardiography and cerebral embolic protection, primary vascular access, choice of guiding catheter, microcatheter, snare, traversal wire, and balloon size.

Preoperative echocardiographic assessment was performed according to latest international guidelines. Collected parameters included left ventricular end-diastolic diameter, left ventricular ejection fraction, aortic valve area, aortic valve peak, and mean pressure gradient. Other valvular lesions were graded as 0 (none/trivial), 1+ (mild), 2+ (moderate), and 3+ (severe). Preoperative electrocardiography-gated computed tomography analysis was performed according to international recommendations, measuring coronary height, valve to coronary, and valve to sinotubular junction. Procedure details including mode of anesthesia, use of transesophageal echocardiography and cerebral protection system, primary vascular access, choice of guiding catheter, microcatheter, snare, traversal wire, and balloon were documented.

### Outcomes

The primary outcome was procedural success, defined as successful leaflet traversal, leaflet modification, and transcatheter heart valve implantation without coronary obstruction, emergent cardiovascular surgery, stroke, or mortality in 30 days. Secondary outcomes were defined per VARC-3 (Valve Academic Research Consortium-3) criteria at 30 days^17^: all-cause mortality, cardiovascular and noncardiovascular mortality, coronary obstruction, stroke, intracranial hemorrhage, cardiac tamponade, aortic root rupture, acute kidney injury (stages 2-3), vascular complications, emergent cardiac surgery, and major bleeding (VARC grade ≥3). Functional status was defined by NYHA functional classification.

### Statistical analysis

Categorical variables were described as frequency (percentage). Continuous variables were tested for normality using the Shapiro-Wilk test and described as mean ± SD or median (IQR). For all variables, the number of patients with available data out of the total is reported to transparently describe missingness; as the extent of missing data was not significant for key outcomes and echocardiographic parameters, and results are presented descriptively without between-group comparisons, no imputation or sensitivity analyses were performed. Comparisons between groups were performed using chi-square test, Fisher's exact test, Student's *t*-test, Welch's *t*-test, and Mann-Whitney *U* test. *P* values <0.05 were considered statistically significant. Analyses were performed using Python version 3.9.5 with packages pandas version 2.0.2, numpy version 1.24.3, and scipy version 1.13.1.

## Results

### Baseline characteristics

**Figure 1:**
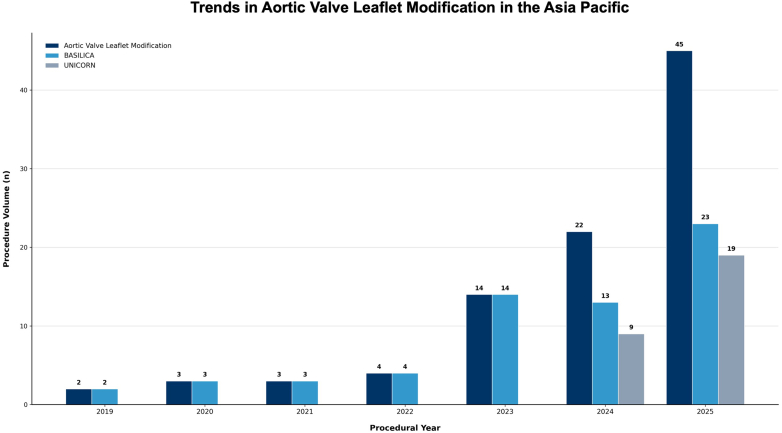
Case Volume of Aortic Valve Leaflet Modification in the Asia-Pacific From 2019 to 2022, annual case volumes ranged from 2 to 4 but between 2023 and 2025, case numbers increased substantially, increasing from 14 to 45 per year. Grey = unilateral UNICORN; Light blue = unilateral BASILICA; Dark blue = all unilateral and bilateral leaflet modification.

A total of 100 patients underwent aortic valve leaflet modification during TAVR between April 2019 and January 2026. Median age was 80 years (76-84) and 72.2% were female. From 2019 to 2022, annual case volumes ranged from 2 to 4 but between 2023 and 2025, case numbers increased substantially, increasing from 14 to 45 per year (Figure 1). Most patients underwent BASILICA (67.0%) and a smaller proportion received UNICORN (30.0%). Three patients underwent bilateral BASILICA-UNICORN. Baseline characteristics of the patients are summarized in Table 1.

**Table 1:** Baseline Characteristics

	Combined (N = 100)	BASILICA (n = 67)	UNICORN (n = 30)	Bilateral BASILICA-UNICORN (n = 3)
Age, y	80.0 (75.0-83.8)	80.0 (76.0-84.0)	77.0 ± 8.56	80.0 (78.5-84.5)
Female Male	57/79 (72.2) 22/79 (27.8)	42/55 (76.4) 13/55 (23.6)	12/21 (57.1) 9/21 (42.9)	3/3 (100) 0/3 (0)
Hypertension	68/99 (68.7)	51/67 (76.1)	15/29 (51.7)	2/3 (66.7)
Diabetes mellitus	42/99 (42.4)	34/67 (50.7)	6/29 (20.7)	2/3 (66.7)
Ischemic heart disease	35/100 (35.0)	20/67 (29.9)	13/30 (43.3)	2/3 (66.7)
Acute coronary syndrome	9/99 (9.09)	6/67 (8.96)	3/29 (10.3)	0/3 (0)
Prior PCI	18/100 (18.0)	14/67 (20.9)	3/30 (10.0)	1/3 (33.3)
Prior CABG	15/100 (15.0)	12/67 (17.9)	3/30 (10.0)	0/3 (0)
Atrial fibrillation	26/100 (26.0)	18/67 (26.9)	8/30 (26.7)	0/3 (0)
Prior CIED	10/100 (10.0)	5/67 (7.46)	5/30 (16.7)	0/3 (0)
Prior stroke/TIA	16/100 (16.0)	14/67 (20.9)	2/30 (6.67)	0/3 (0)
Prior intracranial hemorrhage	1/99 (1.01)	0/67 (0)	1/29 (3.45)	0/3 (0)
COPD	19/99 (19.2)	17/67 (25.4)	1/29 (3.45)	1/3 (33.3)
eGFR (CKD-EPI), mL/min/1.73 m^2^	56.0 ± 23.2	55.7 ± 22.3	56.1 ± 26.5	64.0 (57.3-64.2)
EuroSCORE II	6.92 (4.03-14.0)	7.52 (3.50-14.0)	6.87 (4.89-15.4)	5.50 (5.34-5.80)
Aortic valve area, cm^2^	0.700 (0.600-0.900)	0.690 (0.600-0.900)	0.750 (0.640-0.980)	0.650 (0.590-0.675)
Peak AV gradient, mm Hg	73.0 ± 25.5	75.1 ± 26.6	68.6 ± 24.1	72.0 (70.0-72.0)
Mean AV gradient, mm Hg	45.5 ± 16.4	48.0 ± 16.8	40.3 ± 15.4	40.0 (39.5-42.0)
LVEF, %	62.0 (57.0-69.0)	63.0 (58.5-69.0)	60.0 (55.0-66.8)	69.0 (67.0-76.5)
LVEDD, cm	4.39 ± 0.721	4.31 ± 0.707	4.61 ± 0.738	4.00 (3.95-4.05)
Aortic stenosis grade	1+: 4/74 (5.41) 2+: 4/74 (5.41) 3+: 66/74 (89.2)	1+: 1/43 (2.33) 2+: 2/43 (4.65) 3+: 40/43 (93.0)	1+: 3/28 (10.7) 2+: 2/28 (7.14) 3+: 23/28 (82.1)	3: 3/3 (100)
Aortic regurgitation grade	0: 13/75 (17.3) 1+: 31/75 (41.3) 2+: 22/75 (29.3) 3+: 9/75 (12.0)	0: 4/43 (9.30) 1+: 21/43 (48.8) 2+: 16/43 (37.2) 3+: 2/43 (4.65)	0: 9/29 (31.0) 1+: 8/29 (27.6) 2+: 5/29 (17.2) 3+: 7/29 (24.1)	1: 2/3 (66.7) 2: 1/3 (33.3)
Mitral stenosis grade	0: 80/99 (80.8) 1+: 13/99 (13.1) 2+: 3/99 (3.03) 3+: 3/99 (3.03)	0: 55/67 (82.1) 1+: 10/67 (14.9) 2+: 1/67 (1.49) 3+: 1/67 (1.49)	0: 23/29 (79.3) 1+: 3/29 (10.3) 2+: 1/29 (3.45) 3+: 2/29 (6.90)	0: 2/3 (66.7) 2: 1/3 (33.3)
Mitral regurgitation grade	0: 12/75 (16.0) 1+: 32/75 (42.7) 2+: 24/75 (32.0) 3+: 7/75 (9.33)	0: 8/43 (18.6) 1+: 18/43 (41.9) 2+: 12/43 (27.9) 3+: 5/43 (11.6)	0: 2/29 (6.90) 1+: 13/29 (44.8) 2+: 12/29 (41.4) 3+: 2/29 (6.90)	0: 2/3 (66.7) 1: 1/3 (33.3)
Tricuspid regurgitation grade	0: 10/74 (13.5) 1+: 37/74 (50.0) 2+: 21/74 (28.4) 3+: 6/74 (8.11)	0: 8/43 (18.6) 1+: 21/43 (48.8) 2+: 10/43 (23.3) 3+: 4/43 (9.30)	0: 1/28 (3.57) 1+: 14/28 (50.0) 2+: 11/28 (39.3) 3+: 2/28 (7.14)	0: 1/3 (33.3) 1: 2/3 (66.7)
At-risk coronary height, mm	8.00 (6.33-10.0)	8.20 (6.00-9.97)	8.53 ± 3.18	9.00 (8.00-10.5)
At-risk VTC distance, mm	3.40 (2.35-4.20)	3.00 (2.04-4.00)	4.20 ± 1.39	2.25 (1.73-3.07)

Values are n (%) unless otherwise indicated. Severity of valvular lesions were graded as 0 (none/ trivial), 1+ (mild), 2+ (moderate), and 3+ (severe).

ACS = acute coronary syndrome; AV = aortic valve; BASILICA = bioprosthetic or native aortic scallop intentional laceration to prevent iatrogenic coronary artery obstruction; CABG = coronary artery bypass grafting; CIED = cardiac implantable electronic device; CKD-EPI = Chronic Kidney Disease–Epidemiology Collaboration; COPD = chronic obstructive pulmonary disease; eGFR = estimated glomerular filtration rate; F = female; LVEDD = left ventricular end-diastolic diameter; LVEF = left ventricular ejection fraction; M = male; PCI = percutaneous coronary intervention; TIA = transient ischemic attack; UNICORN = undermining iatrogenic coronary obstruction with radiofrequency needle; VTC = valve-to-coronary.

### Index and second valve characteristics

**Figure 2:**
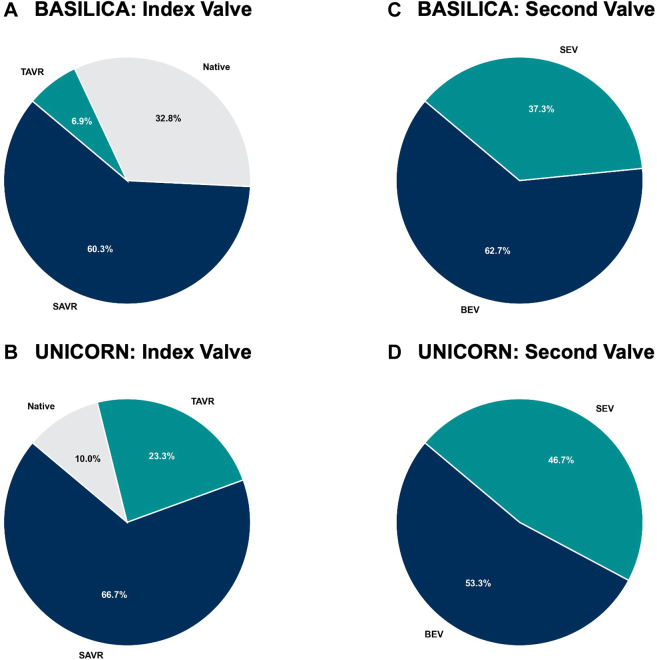
Distribution of Index and Second Valves Index valves for (A) bioprosthetic or native aortic scallop intentional laceration to prevent iatrogenic coronary artery obstruction (BASILICA) and (B) undermining iatrogenic coronary obstruction with radiofrequency needle (UNICORN). Second valves for (C) BASILICA and (D) UNICORN. BEV = balloon-expandable valve; SEV = self-expanding valve; SAVR = surgical aortic valve replacement; TAVR = transcatheter aortic valve replacement.

Among patients treated with BASILICA, the index valve was a surgical aortic valve (SAV) in 60.3%, native in 32.8%, and TAVR in 6.9%. In the UNICORN group, the index valve was SAV in 66.7%, native in 10.0%, and TAVR in 23.3% (Figure 2). Index valves were notably small: 66.7% of Perimount valves were size 19 or 21 mm; 63.6% of Mitroflow valves were size 19 or 21 mm; 66.7% of Trifecta valves were size 19 or 21 mm; and 66.7% of SAPIEN platform valves were size 23 mm (Table 2). A detailed distribution of index valves is provided in Supplemental Table 1.

**Table 2:** Index Valve (SAV)

	Size	
Perimount^a^	19	4/12 (33.3)
	21	4/12 (33.3)
	23	2/12 (16.7)
	25	2/12 (16.7)
Mitroflow	19	3/11 (27.3)
	21	4/11 (36.4)
	23	2/11 (18.2)
	25	1/11 (9.10)
	27	1/11 (9.10)
Trifecta	19	9/27 (33.3)
	21	9/27 (33.3)
	23	4/27 (14.8)
	25	5/27 (18.5)
Index valve (TAV)^b^	Size	
Evolut^c^	23	1/3 (33.3)
	29	2/3 (66.7)
SAPIEN^d^	23	4/6 (66.7)
	26	1/6 (16.7)
	29	1/6 (16.7)

Values in parentheses are percentages.

TAV = transcatheter aortic valve prosthesis; SAV = surgical aortic valve prosthesis.

a Perimount group includes Perimount, Perimount Magna, and Perimount Magna Ease.

b Full list of index valves is available in the Supplemental Materials.

c Evolut group includes Evolut R and Evolut Pro.

d SAPIEN group includes SAPIEN XT and SAPIEN 3.

The second valve implanted in BASILICA patients was a balloon-expandable valve (BEV) in 62.7% and a self-expanding valve in 37.3%. In the UNICORN group, second valves were BEV in 53.3% and self-expanding valve in 46.7%, respectively. The second valves were also small, with 82.9% of Evolut platform valves being size 23 mm, and 79.3% of SAPIEN platform valves being size 20 or 23 mm (Table 3). A detailed distribution is provided in Supplemental Table 2.

**Table 3:** Second Valve (BEV)

SAPIEN^a^	20	21/58 (36.2)
	23	25/58 (43.1)
	26	11/58 (19.0)
	29	1/58 (1.70)
MyVal	20	2/3 (66.7)
	23	1/3 (33.3)
Second valve (SEV)^b^	Size (mm)	
Evolut^c^	23	29/35 (82.9)
	26	6/35 (17.1)
Navitor	23	1/4 (25.0)
	25	3/4 (75.0)

Values in parentheses are percentages.

BEV = balloon-expandable valve; SEV = self-expanding valve.

a SAPIEN group includes SAPIEN 3, SAPIEN 3 Ultra, and SAPIEN 3 Ultra Resilia.

b Full list of second valves is available in the Supplemental Materials.

c Evolut group includes Evolut R, Evolut Pro, Evolut FX, and Evolut FX+.

### Procedural characteristics, leaflet modification tools, and techniques

Geneal anesthesia and transesophageal echocardiography were used in all patients. All patients had transfemoral TAVR, with the exception of 1 patient who had transcarotid TAVR; 96.5% patients had cerebral embolic protection. Target leaflet was left-sided in 87.0%, right-sided in 10.0%, and bilateral in 3.0%. There was a trend toward more right-sided leaflet modification in patients treated with UNICORN than BASILICA (20.0% vs 6.0%, *P* < 0.001) (Supplemental Figure 1).

**Figure 3:**
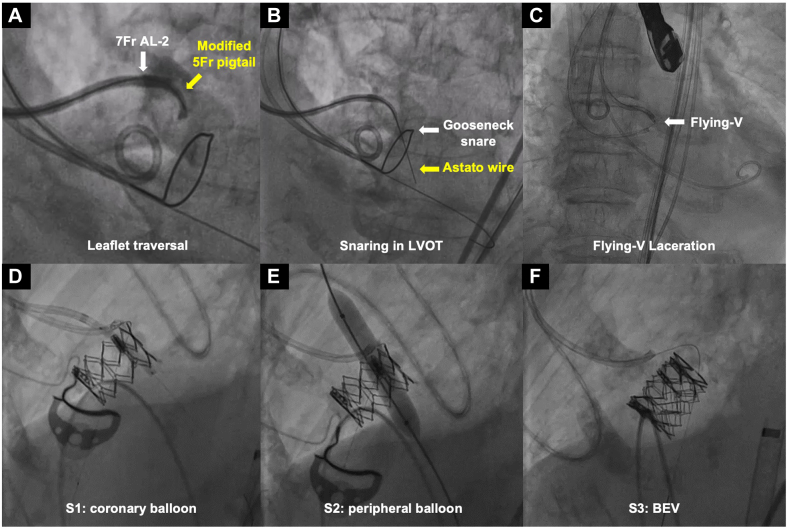
Innovation and Regional Adaptation of BASILICA (A-C) Mother-and-child guiding catheter with AL-2 and manually modified pigtail in a case of BASILICA. (D-F) The 3 key steps in UNICORN technique with BEV include dilatation of the fenestration with coronary (S1) and peripheral balloon (S2), followed by insertion of the BEV into the fenestration for intraleaflet deployment (S3). LVOT = left ventricular outflow tract; other abbreviations as in Figure 2.

For BASILICA patients, a mother-and-child telescoping system was used as guiding catheter in 55.1% of patients. Commonly involving AL1/2 with 5-F long pigtail catheter with manually modified tip (43.5%) (Figures 3A to 3C), AL3 was uncommonly used in this cohort (2.90%), likely reflecting intrinsically small aortic root. The most commonly used microcatheter was Finecross (73.1%). Astato XS20 was used as traversal wire in all patients. Left ventricular outflow tract goose neck snares were 15 mm (7.30%), 20 mm (61.8%), and 25 mm (21.8%), respectively. Balloon-assisted BASILICA^18^ was performed in 15 patients (22.4%). Coronary anchor wire technique was used in 2 patients (2.99%) (Table 4, Figures 3D-3F).

**Table 4:** BASILICA Tools

Category	Tool	
Guiding catheter	AL0.75-Pigtail	1/69 (1.40)
	AL1	15/69 (21.7)
	AL1-IMA	1/69 (1.40)
	AL1-JR3.5	2/69 (2.90)
	AL1-Pigtail	23/69 (33.3)
	AL1.5	1/69 (1.40)
	AL2	10/69 (14.5)
	AL2-JR3.5	1/69 (1.40)
	AL2-MPA	1/69 (1.40)
	AL2-Pigtail	7/69 (10.1)
	AL3	2/69 (2.90)
	EBU4-IMA	1/69 (1.40)
	JR3.5	1/69 (1.40)
	JR4	1/69 (1.40)
	SAL-Pigtail	1/69 (1.40)
	SAL1	1/69 (1.40)
Microcatheter	Caravel	7/52 (13.5)
	Corsair	3/52 (5.80)
	Finecross	38/52 (73.1)
	Hightrack 1.5	1/52 (1.90)
	NaviCross	1/52 (1.90)
	Piggyback	2/52 (3.80)
Traversal wire	Astato XS20	70/70 (100.0)
LVOT snare	15 mm	4/55 (7.30)
	20 mm	34/55 (61.8)
	25 mm	12/55 (21.8)
	30 mm	1/55 (1.80)
	35 mm	4/55 (7.30)

Values in parentheses are percentages.

LVOT = left ventricular outflow tract; other abbreviation as in Table 1.

Among UNICORN patients, the most commonly used guiding catheters were AL1 (66.7%) and AL2 (15.2%), respectively. NaviCross as a microcatheter was used in 80.0%. Traversal wire was VersaCross J-Tip in 57.6% and Astato XS20 in 42.4% (Supplemental Table 3).

### Procedural outcome, in-hospital, and 30-day clinical outcomes

**Figure 4:**
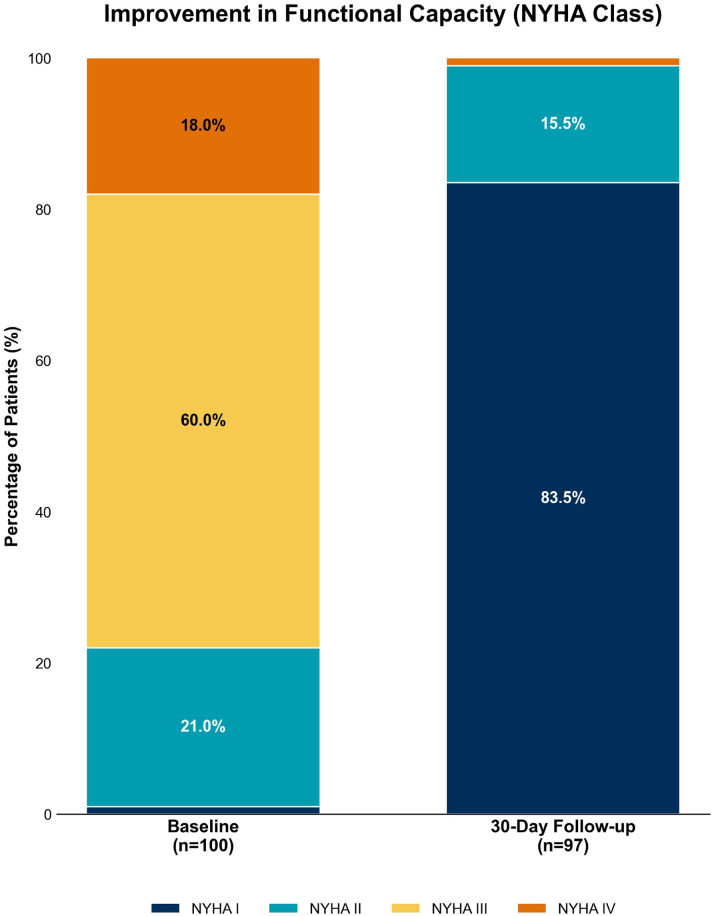
Change in Functional Status Functional status significantly improved after operation, with 84.0% of patients achieving NYHA functional class I at 30 days (*P* < 0.001) in the whole cohort.

In the BASILICA group, 91.0% of patients had successful leaflet traversal, 89.6% had successful leaflet laceration. The procedural success rate was 85.1%, with 3 intraprocedural complications (4.48%), including 2 patients who experienced coronary obstruction (2.99%), with one requiring bail-out stenting and the other emergent conversion to open surgery, and 1 patient developed acute mitral regurgitation requiring emergent cardiac surgery. In addition, vascular complications occurred in 3 patients (4.48%) and acute kidney injury in 1 patient (1.49%). All 7 patients with unsuccessful leaflet laceration did not eventually develop coronary obstruction. At 30 days, noncardiovascular mortality in the BASILICA group was 2.99% (Tables 5 and 6) Functional status significantly improved after operation, with 81.5% of patients achieving NYHA functional class I at 30 days (*P* < 0.001) in the BASILICA group (Figure 4).

**Table 5:** Procedural Outcomes

BASILICA leaflet traversal success	61/67 (91.0)
BASILICA leaflet modification success	60/67 (89.6)
BASILICA procedural success^a^	57/67 (85.1)
UNICORN leaflet traversal success	30/30 (100)
UNICORN leaflet modification success	30/30 (100)
UNICORN procedural success^a^	29/30 (96.7)

Values in parentheses are percentages.

Abbreviations as in Table 1.

a Procedural success was defined as successful target leaflet traversal, leaflet modification, and transcatheter heart valve implantation without mortality, coronary obstruction, stroke, or emergent surgery in 30 days.

**Table 6:** Clinical Outcomes at 30 Days

	Combined (N = 100)	BASILICA (n = 67)	UNICORN (n = 30)	Bilateral BASILICA-UNICORN (n = 3)
Cardiovascular mortality	0 (0)	0 (0)	0 (0)	0 (0)
Noncardiovascular mortality	3 (3.00)	2 (2.99)	1 (3.30)	0 (0)
Ischemic stroke	1 (1.00)	0 (0)	1 (3.30)	0 (0)
Intracranial hemorrhage	1 (1.00)	0 (0)	0 (0)	1 (33.3)
Coronary obstruction	2 (2.00)	2 (2.99)	0 (0)	0 (0)
Cardiac tamponade	1 (1.00)	0 (0)	0 (0)	1 (33.3)
Aortic rupture	0 (0)	0 (0)	0 (0)	0 (0)
AKI (stage 2-3)	3 (3.00)	1 (1.49)	2 (6.70)	0 (0)
Vascular complication	3 (3.00)	3 (4.48)	0 (0)	0 (0)
Emergent CV surgery	2 (2.00)	2 (2.99)	0 (0)	0 (0)
Major bleeding (VARC-3+)	1 (1.00)	0 (0)	1 (3.30)	0 (0)

Values are n (%).

AKI = acute kidney injury; CV = cardiovascular; VARC-3 = Valve Academic Research Consortium 3; other abbreviations as in Table 1.

In the UNICORN group, all patients had successful leaflet traversal and leaflet modification. Procedural success was achieved in 96.7% of patients. During the index hospitalization, ischemic stroke occurred in 1 patient (3.30%) who had a cerebral protection device used, major bleeding (VARC-3+) in 1 patient (3.30%), and acute kidney injury (Stage 2-3) in 2 patients (6.70%). At 30 days, noncardiovascular mortality in the UNICORN group was 3.30% (Tables 5 and 6) Functional status significantly improved after operation, with 89.7% patients achieving NYHA functional class I at 30 days (*P* < 0.001) in the UNICORN group (Figure 4).

Among the 3 patients who received the bilateral BASILICA-UNICORN procedure, 1 patient had intraprocedural pericardial effusion and cardiac tamponade requiring urgent drainage. The same patient also had intracranial hemorrhage during hospitalization. There was no mortality at 30 days (Table 6).

### Echocardiographic outcomes

Predischarge echocardiography showed that 18.2% and 17.2% of patients from BASILICA and UNICORN groups, respectively, had mild aortic regurgitation or above. At 30 days, patients in the BASILICA group had relatively high median aortic valve mean gradient of 15.0 mm Hg [11.0–19.5] (Table 7).

**Table 7:** Echocardiographic findings

	BASILICA	UNICORN
Preoperative		
AS grade	1: 1/43 (2.33) 2: 2/43 (4.65) 3: 40/43 (93.0)	1: 3/28 (10.7) 2: 2/28 (7.14) 3: 23/28 (82.1)
AVA, cm^2^	0.690 (0.600-0.900)	0.750 (0.640-0.980)
Peak AV gradient, mm Hg	75.1 ± 26.6	68.6 ± 24.1
Mean AV gradient, mm Hg	48.0 ± 16.8	40.3 ± 15.4
AR grade	0: 4/43 (9.30) 1: 21/43 (48.8) 2: 16/43 (37.2) 3: 2/43 (4.65)	0: 9/29 (31.0) 1: 8/29 (27.6) 2: 5/29 (17.2) 3: 7/29 (24.1)
MS grade	0: 55/67 (82.1) 1: 10/67 (14.9) 2: 1/67 (1.49) 3: 1/67 (1.49)	0: 23/29 (79.3) 1: 3/29 (10.3) 2: 1/29 (3.45) 3: 2/29 (6.90)
MR grade	0: 8/43 (18.6) 1: 18/43 (41.9) 2: 12/43 (27.9) 3: 5/43 (11.6)	0: 2/29 (6.90) 1: 13/29 (44.8) 2: 12/29 (41.4) 3: 2/29 (6.90)
TR grade	0: 8/43 (18.6) 1: 21/43 (48.8) 2: 10/43 (23.3) 3: 4/43 (9.30)	0: 1/28 (3.57) 1: 14/28 (50.0) 2: 11/28 (39.3) 3: 2/28 (7.14)
LVEF, %	63.0 (58.5-69.0)	60.0 (55.0-66.8)
LVEDD, cm	4.31 ± 0.707	4.61 ± 0.738
Postoperative (predischarge)		
Peak AV gradient, mm Hg	21.7 ± 12.6	20.5 (15.5-24.2)
Mean AV gradient, mm Hg	12.0 ± 6.78	10.0 (7.00-11.0)
AR grade	0: 54/66 (81.8) 1: 12/66 (18.2)	0: 24/29 (82.8) 1: 4/29 (13.8) 2: 1/29 (3.45)
Postoperative (1 month)		
Peak AV gradient, mm Hg	27.6 ± 12.5	20.5 ± 7.85
Mean AV gradient, mm Hg	15.0 (11.0-19.5)	10.0 (9.00-13.0)
AR grade	0: 46/51 (90.2) 1: 3/51 (5.88) 2: 2/51 (3.92)	0: 22/25 (88.0) 1: 2/25 (8.00) 2: 1/25 (4.00)
MS grade	0: 41/51 (80.4) 1: 7/51 (13.7) 2: 3/51 (5.88)	0: 20/24 (83.3) 1: 2/24 (8.33) 2: 1/24 (4.17) 3: 1/24 (4.17)
MR grade	0: 9/27 (33.3) 1: 14/27 (51.9) 2: 3/27 (11.1) 3: 1/27 (3.70)	0: 4/23 (17.4) 1: 15/23 (65.2) 2: 2/23 (8.70) 3: 2/23 (8.70)
TR grade	0: 3/27 (11.1) 1: 17/27 (63.0) 2: 4/27 (14.8) 3: 3/27 (11.1)	0: 2/23 (8.70) 1: 16/23 (69.6) 2: 4/23 (17.4) 3: 1/23 (4.35)
LVEF, %	60.8 ± 7.29	63.5 ± 10.1
LVEDD, cm	4.20 ± 0.693	4.27 ± 0.683

Values are n (%) unless otherwise indicated. Severity of valvular lesions were graded as 0 (none/ trivial), 1+ (mild), 2+ (moderate), and 3+ (severe).

AR = aortic regurgitation; AS = aortic stenosis; AVA = aortic valve area; MR = mitral regurgitation; MS = mitral stenosis; TR = tricuspid regurgitation; other abbreviations as in Table 1.

## Discussion

We report the first multicenter aortic valve leaflet modification registry from the Asia-Pacific region. Our findings demonstrated the following key findings: 1) increasing procedural volume across the region for both BASILICA and UNICORN techniques; 2) procedural success rates were high, and the procedures were generally safe; 3) the cohort was characterized by small anatomy, which influenced the choice of technique, transcatheter tools, and valve selection; and 4) Postprocedural echocardiographic gradients were high perhaps due to the use of small intra-annular valves, although recent publication on medium-term follow-up suggested no clinical outcome differences^19^ (Central Illustration).

**Central Illustration:**
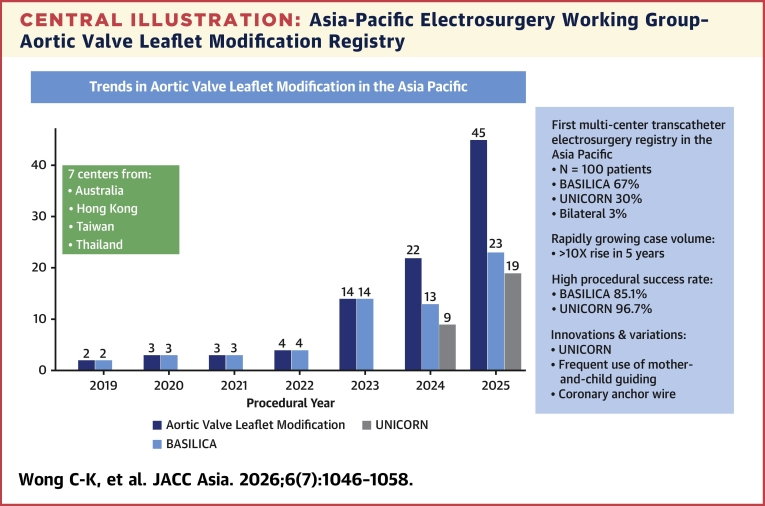
Asia-Pacific Electrosurgery Working Group–Aortic Valve Leaflet Modification Registry The first multicenter transcatheter electrosurgery registry from the Asia-Pacific involving 100 patients from 7 centers from Australia, Hong Kong, Taiwan, and Thailand. Rapidly increasing case volume and acceptable short-term outcome were observed. BASILICA = bioprosthetic or native aortic scallop intentional laceration to prevent iatrogenic coronary artery obstruction; UNICORN = undermining iatrogenic coronary obstruction with radiofrequency needle; Grey = unilateral UNICORN; Light blue = unilateral BASILICA; Dark blue = all unilateral and bilateral leaflet modification.

### Unique aspects of the Asia-Pacific region on leaflet modification in TAVR

Since the initial introduction of BASILICA, leaflet modification using transcatheter electrosurgery has been widely adopted in North America and Europe. Both prospective trials and real-world data have demonstrated the safety and efficacy of the technique.^12^^,^^13^^,^^20^ However, Asian populations have been underrepresented in these studies. Given that Asian individuals have distinct anatomic features, including small aortic root,^21^ and in certain ethnic groups, a higher proportion of bicuspid aortic valves,^22^ it remains uncertain whether similar effectiveness of electrosurgical leaflet modification can be achieved in the Asia-Pacific region. Furthermore, although the UNICORN technique originated in this region, existing case series have not included centers from diverse geographical areas. To address this gap and gather real-world data on transcatheter electrosurgery, the Asia-Pacific Electrosurgery Working Group commissioned the present study.

### Growing case volume on leaflet modification in the Asia-Pacific

As previously implanted valve prostheses degenerate over time, the need for coronary protection during subsequent valve-in-valve procedures has increased.^23^ In the Asia-Pacific region, our registry shows that the adoption of aortic valve leaflet modification has markedly increased, with case volumes expanding nearly 10-fold over the past 5 years (Figure 1). A recently published prediction model by Ohno et al^24^ suggested a major future surge in TAV-in-SAV and TAV-in-TAV in the Asia-Pacific similar to previous prediction algorithm in the United States.^25^ Redo TAVR presents specific challenges due to heterogeneity of index TAVR placement, types (supra-annular vs intra-annular), and overall higher coronary obstruction risk than TAVR in surgical prostheses.^26^ All these challenges are amplified in the Asia-Pacific regions due to anatomical constraints, as Asian individuals are reported to have small aortic roots and narrower, lower sinotubular junctions, all of which contribute to a higher risk of coronary obstruction.^21^^,^^27^ Consequently, leaflet modification, which disrupts leaflet anatomy to maintain patent coronary flow, is becoming a critical step in valve-in-valve procedures. Currently, electrosurgical leaflet laceration using BASILICA is widely employed in the region, while UNICORN is emerging as an alternative strategy.

### Regional innovation and adaptation of technique

The UNICORN technique was developed and first performed in the region in 2022 successfully in a TAV-in-TAV case.^7^ The technique involves traversal of the target leaflet with electrified wire, followed by serial dilatation of the fenestration using coronary and peripheral balloon. Subsequently, BEV is inserted into the fenestration. When the BEV is inflated and deployed, it simultaneously opens the target leaflet and pushes the disrupted leaflets away from the coronary ostium. The technique had since been adopted worldwide. Similar to a recently published single-center UNICORN registry,^9^ a high procedural success rate was observed in this multicenter registry. Although a small number of native valve cases were successfully treated in this cohort, operators should exercise caution when considering leaflet modification in this setting given the limited data regarding safety and efficacy.

**Figure 5:**
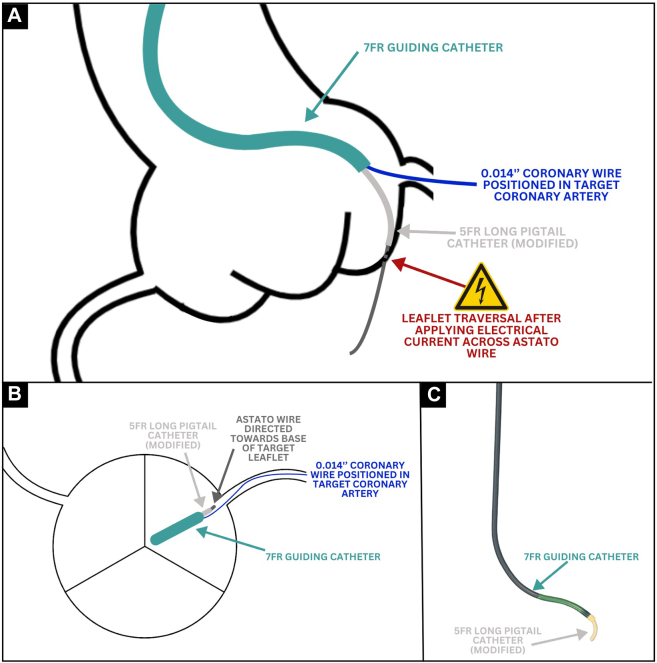
Unique Strategies Used in the Asia-Pacific Region for BASILICA (A and B) Mother-and-child system comprising 7-F guide, 5-F long pigtail with manually amputated tip, and Astato XS20 wire. (C) Coronary ostium anchoring technique with 7-F guide, 0.014-inch wire in the target coronary artery, and 5-F long pigtail with manually amputated tip and Astato XS20 wire for leaflet traversal. Abbreviation as in Figure 2.

BASILICA remains the most commonly used technique for aortic leaflet modification in the Asia-Pacific region. Several notable observations emerged from our registry. First, in contrast to operators in North America and Europe, who frequently report successful outcomes using an AL3 guiding catheter, this catheter was rarely utilized in our study. This disparity is likely due to small patient anatomy and the limited availability of this catheter in many catheterization laboratories. Second, given that aortic roots are generally small in our population, standalone guiding catheters such as AL1 or AL2 often fail to reach the basal leaflet with sufficient inward angulation. Consequently, more than half of the patients in this cohort were treated using mother-and-child systems. These setups typically involved an AL1 or AL2 guide combined with an amputated pigtail catheter to enable inward angulation and coaxial contact with the leaflet of interest.^28^ This registry represents the largest cohort to date utilizing this approach, providing valuable data on its reliability. Finally, another innovative technique developed in the region is the coronary ostium anchoring method,^28^ in which a coronary wire adjacent to a pigtail catheter is inserted into the coronary artery of interest. This theoretically ensures that the final leaflet laceration is positioned directly in front of the coronary ostium (Figure 5).

### Leaflet modification technique with consistent efficacy across geographical regions

Consistent with other international registries on leaflet modification, our data demonstrate high procedural success rates and low coronary obstruction rates. Procedural success was achieved in 85.1% of patients treated with BASILICA in our registry, comparable to the 86.9% reported in the International BASILICA registry^12^ and the 88.2% in the EURO BASILICA registry.^13^ The coronary obstruction rate in our registry was 2.99%, comparable to 4.7% in the International BASILICA registry and 2.4% in the EURO BASILICA registry. Our study similarly demonstrates effective coronary protection.^9^ Overall, the most challenging step of BASILICA was leaflet traversal, with 8.96% attempts being unsuccessful. In contrast, for the remaining 61 patients with successful leaflet traversal, only 1 patient had unsuccessful laceration with BASILICA.

### Future perspectives

There is currently no dedicated device for leaflet modification available in the region. The current registry has suggested a need for devices in the region. In addition, formalized teaching in the technique will be necessary to ensure safe roll-out of the procedure in new centers.

### Study limitations

First, the study included only Asia-Pacific centers with relatively greater experience in leaflet modification. Real-world performance in centers that are newly initiating transcatheter electrosurgery programs may have different outcomes, given the learning curve involved. Second, this registry includes patients with a wide range of clinical scenarios, including native, TAVR-in-SAV replacement, and TAVR-in-TAVR. As the mechanism and risk of coronary obstruction in these 3 distinct scenarios is different, larger studies will be required to evaluate if there are any differences in native vs valve-in-valve procedures. This heterogeneity, although reflective of real-world practice, introduces potential confounding that should be considered when interpreting the aggregated outcomes. Third, the baseline differences between the BASILICA and UNICORN groups preclude any direct comparative conclusions, and findings are best interpreted as descriptive feasibility data for each technique. Fourth, this study reported only short-term outcomes. Follow-up studies will be needed to assess the long-term outcomes in this population. Fifth, this study did not include patients who received other coronary protection strategies such as chimney or snorkel stenting. Further studies are required to compare their relative efficacy. Sixth, the overall sample size is modest and key safety events such as coronary obstruction and mortality were infrequent; readers should therefore interpret these point estimates with appropriate caution, as the precision of the observed rates is limited. Finally, although missing data were infrequent and fully reported, a small residual risk of bias from listwise deletion cannot be entirely excluded.

## Conclusions

In this first multicenter aortic valve leaflet modification registry from the Asia-Pacific region, transcatheter electrosurgical leaflet modification techniques are associated with acceptable short-term outcome in high-risk patients undergoing TAVR in the Asia-Pacific region.

## Funding Support and Author Disclosures

Dr Yin is a proctor for Medtronic and Edwards Lifesciences. Dr Lee is a proctor for Medtronic and Edwards Lifesciences. Dr So is a clinical proctor for Abbott Vascular, Edwards Lifesciences, and Medtronic. Dr Tang has received speaker honoraria and served as a physician proctor, consultant, advisory board member, TAVR publications committee member, RESTORE study steering and screening committee member, APOLLO trial screening committee member, and IMPACT MR steering committee member for Medtronic; has received speaker honoraria and served as a physician proctor, consultant, advisory board member, ENVISION trial screening committee member, and TRILUMINATE trial anatomic eligibility and publications committee member for Abbott Structural Heart; and has served as an advisory board member for Boston Scientific, Anteris, Philips, Edwards Lifesciences, Peija Medical, and Shenqi Medical Technology. Dr Poon is a physician proctor, consultant to Edwards Lifesciences, advisory board member and equity holder of Anteris, and has received speaker honoraria for Edwards Lifesciences and Medtronic. All other authors have reported that they have no relationships relevant to the contents of this paper to disclose.
