# Supplementary material for: Multicenter Experience of Aortic Valve Leaflet Modification in TAVR: From the Asia-Pacific Electrosurgery Working Group
Source: JACC Asia. 2026 Jun 24;6(7):1046–58. doi: 10.1016/j.jacasi.2026.04.017 (PMC13350379; doi:10.1016/j.jacasi.2026.04.017)
Supplement: Supplemental Table 1 to 3 and Supplemental Figure 1 [file mmc1.docx]

**Multicenter Experience of Aortic Valve Leaflet Modification in TAVR: From the Asia-Pacific Electrosurgery Working Group**

^#^Chun-Ka WONG, MBBS;^1,2^ ^#^Simon Cheung-Chi LAM, MBBS;^1,2^ Wei-Hsian YIN, MD;^3,4^ Yung-Tsai Lee, MD;^3,5^ Nattawut WONGPRAPARUT, MD;^6^ Ronen Gurvitch, MBBS;^7^ Mann CHANDAVIMOL, MD;^8^ Kent Chak-Yu SO, MBChB;^9,10^ Ka-Chun UN, MBBS MRes(Med);^1,2^ Ho-On Alston Conrad CHIU, MBBS;^1,2^ Tien-Ping TSAO, MD;^3,11^ Matthew BROOKS, MBBS;^7^ Tawai NGERNSRITRAKUL, MD;^8^ Leo Ka-Lok LAI, MBChB;^9^ Kevin Ka-Ho KAM, MBChB;^9^ Daniel Tai-Leung CHAN, MBBS;^12,13^ Gilbert H. L. TANG, MD MSc MBA;^14^ ^##^Kwong-Yue Eric CHAN, MBBS;^1,2,15^ ^##^Karl POON, MBBS.^16,17^

^1^ Cardiology Division, Department of Medicine, School of Clinical Medicine, Li Ka Shing Faculty of Medicine, The University of Hong Kong, Hong Kong.

^2^ Cardiology Division, Department of Medicine, Queen Mary Hospital, Hong Kong.

^3^ Heart Center, Cheng Hsin General Hospital, Taipei, Taiwan.

^4^ Faculty of Medicine, School of Medicine, National Yang Ming Chiao Tung University, Hsinchu, Taiwan.

^5^ Department of Exercise and Health Science, National Taipei University of Nursing and Health Sciences

^6^ Department of Medicine, Siriraj Hospital, Mahidol University, Bangkok, Thailand.

^7^ Department of Cardiology, Royal Melbourne Hospital, Australia.

^8^ Department of Medicine, Ramathibodi Hospital, Mahidol University, Bangkok, Thailand

^9^ Division of Cardiology, Department of Medicine and Therapeutics, Prince of Wales Hospital, The Chinese University of Hong Kong, Hong Kong.

^10^ Li Ka Shing Institutes of Health Science, The Chinese University of Hong Kong, Hong Kong.

^11^ Faculty of Medicine, National Defense Medical University, Taiwan.

^12^ Department of Surgery, School of Clinical Medicine, Li Ka Shing Faculty of Medicine, The University of Hong Kong, Hong Kong.

^13^ Department of Cardiothoracic Surgery, Queen Mary Hospital, Hong Kong.

^14^ Department of Cardiovascular Surgery, Mount Sinai Health System, New York, New York, USA.

^15^ Cardiac Medical Unit, Grantham Hospital, Hong Kong.

^16^ University of Queensland, Brisbane, Australia.

^17^ The Prince Charles Hospital, Metro North Health, Brisbane, Australia.

^#^Co-first authors

^##^Co-senior authors

**SUPPLEMENTAL FIGURE**

**Supplemental Figure S1. Distribution of target leafet.** The majority of target leaflets were left coronary cusp (LCC) in both (A) BASILICA and (B) UNICORN.  **Abbreviations:** RCC, right coronary cusp.

**
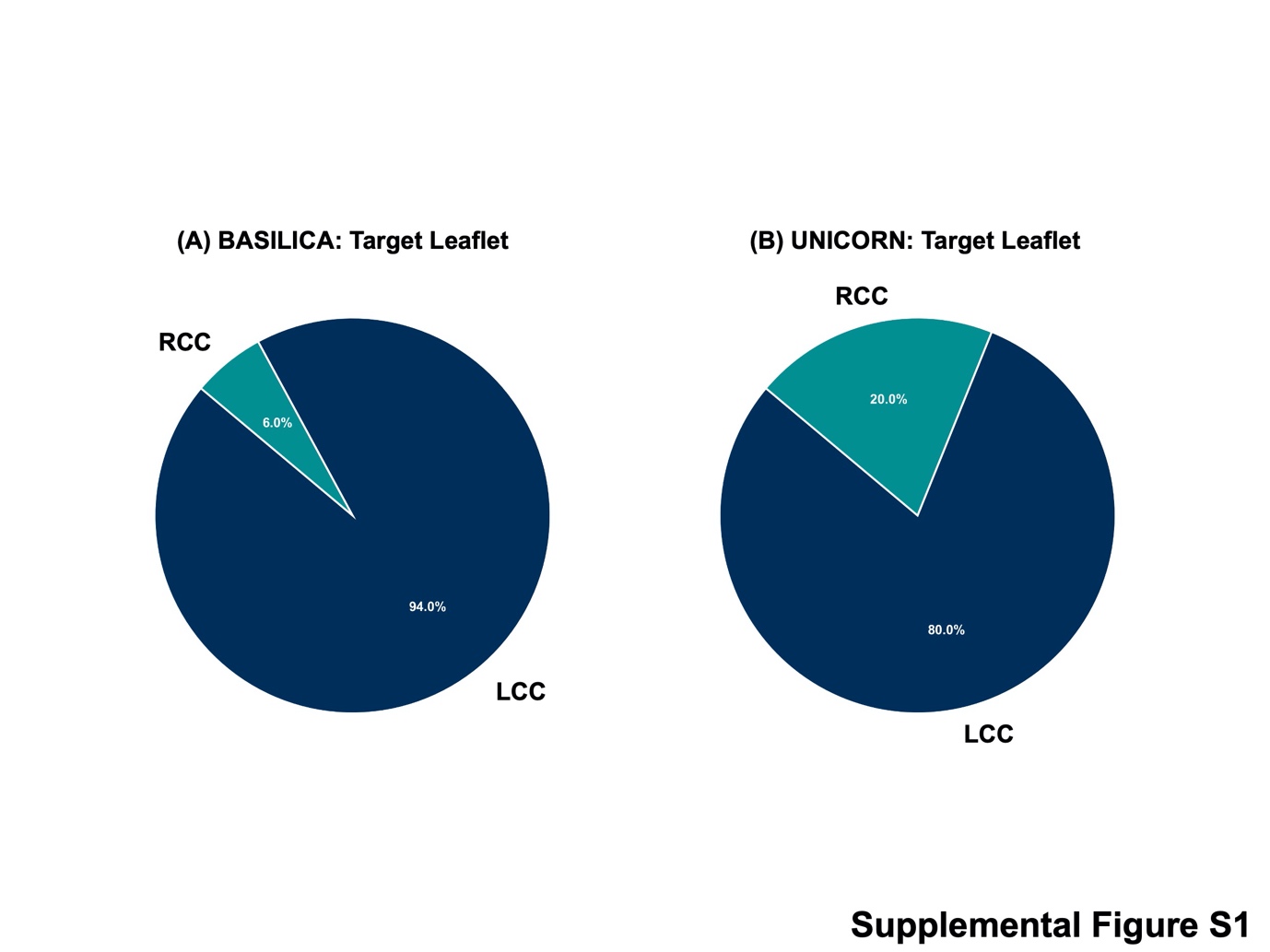
**

**Supplemental Table S1. Index valve**

| **Native** | |
| --- | --- |
| Native | 24/91 (26.4%) |
| **Surgical aortic valve replacement (SAVR)** | |
| Trifecta | 27/91 (29.7%) |
| Mitroflow | 11/91 (12.1%) |
| Perimount Magna Ease | 5/91 (5.5%) |
| Perimount | 4/91 (4.4%) |
| Perimount Magna | 4/91 (4.4%) |
| Epic | 3/91 (3.3%) |
| Perceval S Small | 1/91 (1.1%) |
| **Transcatheter aortic valve replacement (TAVR)** | |
| Sapien 3 | 4/91 (4.4%) |
| CoreValve | 2/91 (2.2%) |
| Evolut Pro | 2/91 (2.2%) |
| Sapien XT | 2/91 (2.2%) |
| ACURATE neo | 1/91 (1.1%) |
| Evolut R | 1/91 (1.1%) |

**Supplemental Table S2. Second valve implanted during TAVR**

| **Balloon expandable valve (BEV)** | |
| --- | --- |
| MyVal | 3/100 (3.00%) |
| SAPIEN 3 | 24/100 (24.0%) |
| SAPIEN 3 Ultra | 11/100 (11.0%) |
| SAPIEN 3 Ultra Resilia | 23/100 (23.0%) |
| **Self-expanding valve (SEV)** | |
| Evolut FX | 15/100 (15.0%) |
| Evolut FX+ | 12/100 (12.0%) |
| Evolut Pro | 4/100 (4.00%) |
| Evolut R | 4/100 (4.00%) |
| Navitor | 4/100 (4.00%) |

**Supplemental Table S3. UNICORN leaflet traversal tools**

| **Category** | **Tool** |  |
| --- | --- | --- |
| Guiding catheter | AL1 | 22/33 (66.7%) |
|  | AL1-JR3.5 | 1/33 (3.00%) |
|  | AL1-Pigtail | 1/33 (3.00%) |
|  | AL2 | 5/33 (15.2%) |
|  | JR4 | 1/33 (3.00%) |
|  | MB | 1/33 (3.00%) |
|  | MPA | 2/33 (6.10%) |
| Microcatheter | Caravel | 2/25 (8.00%) |
|  | Finecross | 3/25 (12.0%) |
|  | NaviCross | 20/25 (80.0%) |
| Traversal wire | Astato XS | 14/33 (42.4%) |
|  | VersaCross J-tip | 19/33 (57.6%) |
